# Geospatial Analysis of the Proportion of Persons Defined as Underrepresented in Medicine for Each Medical School and Their Surrounding Core-Based Statistical Area

**DOI:** 10.1089/heq.2023.0221

**Published:** 2024-02-28

**Authors:** Zoel A. Quiñónez, Angel Benitez-Melo, Laura M. Diaz, Michael Lennig, Danton Char, Charlotte Smith

**Affiliations:** ^1^ Department of Anesthesiology, Perioperative and Pain Medicine, Stanford University School of Medicine, Stanford, California, USA.; ^2^ Graduate Division, School of Public Health, University of California, Berkeley, Berkeley, California, USA.; ^3^ Department of Biology, University of San Diego, San Diego, California, USA.; ^4^ Division of Environmental Health Sciences, School of Public Health, University of California Berkeley, Berkeley, California, USA.

**Keywords:** health equity, diversity, medical education, geospatial analysis, antiracism

## Abstract

**Background:**

The current approach to increasing diversity in medical education fails to consider local community demographics when determining medical school matriculation.

**Purpose:**

We propose that medical schools better reflect their surrounding community, both because racially/ethnically concordant physicians have been shown to provide better care and to repair the historical and current racist impacts of these institutions that have criminalized, displaced, and excluded local Black and Brown communities.

**Methods and Results:**

In this study, we used geospatial analysis to determine that medical school enrollments generally fail to reflect their surrounding community, represented as their core-based statistical area, within which the individual medical schools reside.

## Background

The drive toward diversity in medical education exists in a broad contextual vacuum. It does not approach equity with the intention of repairing the racist impact that the medical industry and individual medical institutions have had on the nation and on local communities.^1–3^ The current strategy of setting general institutional goals that urge schools to focus on diversity or focus on process rather than outcome have predictably failed to yield meaningful gains given the purposeful manner in which the lack of Black and Brown physicians was manufactured.^4,5^

Aside from the structural impacts of colonization, slavery, and segregation, physicians of color were purposefully excluded from White medical schools while medical governing agencies used racist criteria (e.g., Flexner Report) to close Black medical schools, and later used the veil of fiscal prudency to strip these communities of public health services.^2,3,6,7^ In addition, regional antiaffirmative action laws have hampered well-intentioned efforts like the AAMC's Project 3000 by 2000 plan to increase Black and Brown matriculation.^8,9^ Along with the emerging evidence that patients from marginalized communities are better served by physicians from a community with which the patient self-identifies, the need to repair this purposeful exclusion should drive specific strategies to increase the number of Black and Brown physicians.^10–13^

In addition, the complicity of universities and their medical institutions in driving systematic displacement, marginalization, under-resourcing, and criminalization of Black and Brown communities within their proximity should also compel these institutions to independently respond to their own communities' need for educational attainment and for Black and Brown health professionals.^14–16^ For instance, academic institutions continue to use local municipal funds, state and federal grants, and tools like imminent domain to displace, price out, and intimidate members of adjacent existing Brown and Black neighborhoods while leasing out their own university's nonprofit status to biomedical entities to profit from the resultant innovations.^14^

Analyzing how these institutions reflect their local community can help set targets for student cohorts more reflective of the communities they impact and serve, particularly given the evidence that physicians tend to practice within the same regions where they trained.^17^ It can also add to the broader national and state-level data that instruct equity-centered efforts to address the lack of enrolled medical students from marginalized and racialized groups.

Some universities have begun to address institutional admission and hiring practices. For example, Georgetown University is addressing their own previous use of enslaved labor to build and maintain the university by seeking to offer admission to descendents of these slaves, and the University of California Berkeley—UC San Francisco Joint Medical Program is working to institute antiracism practices into their pedagogy and admissions.^1,15,16,18^ But these efforts are sparse.

In this study, we use the designation for racialized groups marginalized from medical education, underrepresented in medicine (URiM), to represent persons from Black, Latina(o)/Hispanic, or Native American communities.^19^ We hypothesized that across medical schools, the proportion of students defined as URiM within each U.S. allopathic medical school differs from the proportion of people that fit the definition of URiM within the surrounding core-based statistical area (CBSA), a designation for a metropolitan area and the surrounding areas connected through commuting.^1,20–22^

## Data and Methods

We used URiM to assess how individual institutions represent the historically underserved and excluded members of their surrounding community. The counts and percentages of URiM for allopathic medical schools were obtained from the AAMC table 2021_FACTS_TABLE_B-5.2.

Population data were collected from the U.S. Census 2020 Redistricting Core-Based Statistical Data layer. We chose CBSA as the geographic unit because this broader geographic unit reasonably represents the community that an academic medical institution might serve and includes areas that may have been displaced from the areas adjacent to the university. Comparisons of the proportion of URiM individuals within medical schools taken from AAMC 2021 Table B5.2 and their respective CBSA were made directly in ArcGIS Pro (ESRI, Redlands) using the test of proportions. A Wilcoxon rank-sum test was used to compare percentages of URiM between schools given the nonparametric distribution and to compare percentages of URiM across CBSAs where these medical schools are situated using R (R Core Team, Vienna). Medical schools were spatially joined with their CBSA to display the data. Arrowheads represent whether institutions were above or below their surrounding area at a significance of 0.05.

## Results

**FIG. 1.**
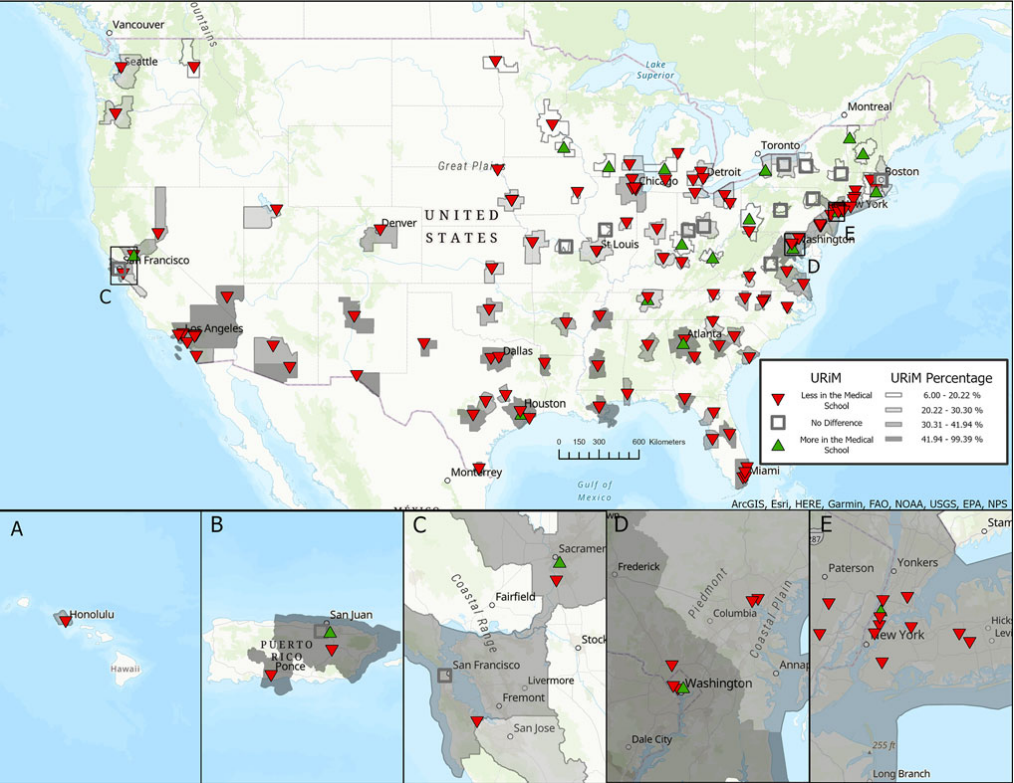
Comparison of the proportion of persons defined as URiM within Medical Schools and their surrounding CBSA. Arrowheads for each medical school depict whether its enrollment of students defined as URiM for the 2021 school year were above (green up triangle), below (red down triangle), or a similar (gray box) categorization within the surrounding CBSA (2020) at a significance level of 0.05. **(A)** Hawaii, **(B)** Puerto Rico, **(C–E)** Areas with a high density of medical schools located within Northern CA, Washington (DC), and New York (NY), respectively. CBSA, core-based statistical area; URiM, underrepresented in medicine.

There were a total of 155 U.S. medical schools listed on the 2021 B5.2 facts table. Of all medical schools, 123 of the 155 (79.4%) had a lower representation of students defined as URiM than the comparable population in their surrounding CBSA, 17 (11%) had a greater proportion than the surrounding area (*p*<0.05), and for 15 (9.7%) schools there was no difference (Fig. 1). There was a higher proportion of persons that would be defined as URiM in CBSA where schools had a lower proportion of URiM compared to those CBSAs where schools had a similar proportion (41.1% vs. 20.9%, *p*=2.04×10^−5^), as well as in those that have lower proportions compared to those that had a higher proportion than their CBSA (41.1 vs. 21.3, *p*=0.007) (Table 1). There was also a higher proportion of URiM in schools with a higher proportion of URiM in comparison to those that had a lower proportion compared to their CBSA (24.7% vs. 19.0%, *p*=0.001) (Table 1).

**Table 1.** Difference in the Median Proportion of Underrepresented in Medicine When Comparing Medical Schools with More, Less, or Similar Proportions to Their Surrounding Core-Based Statistical Area

Comparison group	% Difference between medical schools	^*^p-Value	% Difference between CBSAs	^*^p-Value
Medical Schools > CBSA vs. Medical Schools < CBSA	+5.7 (24.7 vs. 19.0)	0.001	−19.8 (21.3 vs. 41.1)	0.007
Medical Schools > CBSA vs. Medical Schools=CBSA	+4.8 (24.7 vs. 19.9)	0.064	+0.4 (21.3 vs. 20.9)	0.806
Medical Schools < CBSA vs. Medical Schools=CBSA	−0.9 (19.0 vs. 19.9)	0.283	+20.2 (41.1 vs. 20.9)	2.04×10^−5^

^*^ Uncorrected *p*-values.

CBSA, core-based statistical area; URiM, underrepresented in medicine.

There were only six medical schools within a CBSA in the top two quantiles (above the 50th percentile) that had a proportion of URiM individuals above that of their respective CBSA, one of which is in Puerto Rico (Table 2).

**Table 2.** Medical Schools That Reside in a Core-Based Statistical Area in the Top Two Quantiles for the Proportion of Individuals Categorized as Underrepresented in Medicine and That Have a Proportion of Individuals Characterized as Underrepresented in Medicine Either at or Above the Proportion Within Their Respective Core-Based Statistical Area

Medical school	URiM within the medical school (%)	CBSA	URiM within the CBSA (%)	Relationship
City University of New York School of Medicine	46.87	New York – Newark – Jersey City, NY – NJ – PA	41.85	Medical School > CBSA
Howard University College of Medicine	77.87	Washington – Arlington – Alexandria, DC – VA – MD – WV	44.02	Medical School > CBSA
Morehouse School of Medicine	79.33	Atlanta – Sandy Springs – Alpharetta, GA	48.03	Medical School > CBSA
University of California, Davis School of Medicine	36.57	Sacramento – Roseville – Folsom, CA	33.70	Medical School > CBSA
University of California, San Francisco School of Medicine	34.30	San Francisco – Oakland – Berkeley, CA	32.76	No Difference
Universidad Central del Caribe School of Medicine	97.32	San Juan – Bayamón – Caguas, PR	98.98	No Difference
University of Houston School of Medicine	68.33	Houston – The Woodlands – Sugarland, TX	56.49	Medical School > CBSA
University of Puerto Rico School of Medicine	100.00	San Juan – Bayamón – Caguas, PR	98.98	Medical School > CBSA

## Discussion

In 2021, 79.4% of medical schools enrolled a lower proportion of URiM than those that resided within their surrounding communities. Schools with a higher proportion of URiM than their surrounding community admit a higher proportion of URiM compared to those that have a lower proportion. But differences between school performance on reflecting their community seemed to be primarily related to the proportion of persons defined as URiM in the CBSA and less so to a greater enrollment within the medical school. Overall, in 2021 medical schools continue to exclude historically marginalized members of their own communities. Regionally, the Northwest, Great Plains, and Midwest had only one CBSA where a medical school was above the 50th percentile for individuals that could be categorized at URiM. Schools demonstrating a proportion of URiM at or above their respective CBSA in this region likely did so due to a much lower proportion of these individuals in the community.

To achieve equity in medical training and repair racialized exclusion, there is a need for specific, precise, and actionable antiracist strategies like the “racism as a root-cause” approach that has been advocated within public health to create long-term system-wide change toward health equity.^23–25^ The recent Supreme Court decision in *Students for Fair Admission, Inc. versus Presidents and Fellows of Harvard College* preventing the use of race or ethnicity to select students for admission to medical school complicates these efforts.^26^ In California, after the passage of *Proposition 209*, the use of outreach toward economically oppressed communities has had some benefit in mitigating the impact of eliminating race and ethnicity from admissions. However, these strategies have proven inadequate in providing access to higher education for racialized groups subjected to structural racism and the disparate access it creates.^27^

Focusing on eliminating or minimizing the impact of admission policies that lead to inequity, like the use of Medical College Admission Test (MCAT) scores in the admissions process, should be a focus of efforts to increase the admission and enrollment of persons from marginalized racialized groups.^28,29^ Recent studies, for instance, demonstrate that students within the middle third of MCAT scores fair similarly to students with MCAT scores in the upper third but represent a group with a far greater proportion of URiM.^28,29^

In addition, the use of experiences (food/housing insecurity, the need for work study, or even those related to race), as well as place of origin (e.g., zip code, census tract, medically underserved area), multilingualism, and the construction of pipeline programs are potential strategies.^30,31^ Substantive rather than symbolic inclusion within medical school admissions of members from nearby communities that have historically been excluded could serve to bolster the selection of students from these communities. Advocacy against color blind and race-neutral policies is also necessary.^22^

Schools that have succeeded at enrolling URiM physicians in a way that reflects their surrounding community may serve as strategic models toward increasing URiM students in medical education, particularly those in California that have already contended with the elimination of race and ethnicity from admission practices, like UC Davis and UC San Francisco^23^ (Table 2). Finally, residing in a community with a low percentage of persons that would be defined as URiM does not obviate the need for a medical school to serve the national, regional, and local duty to increase representation from these groups given that marginalization has been a local and national process. Schools in areas with a low proportion of URiM should at the least aim for a representation comparable to the national census.

There are several limitations to our analysis that should be addressed in future work. First, we used URiM as a proxy for Black, Hispanic, and American Indian in medicine, which is an imperfect categorization that does not distinguish between these populations nor between people whose families have been in their local communities for generations and those that have not. However, this study serves as a first step in examining the relationship between the student body of medical colleges and the areas within which they are situated. Further analysis of disparities for individual races and ethnicities is needed, as experiences across race and ethnic groups are heterogeneous even when similarly rooted in structural racism. In addition, some schools may focus on access for students with different histories of marginalization (economic, linguistic, geographic, rural–urban divide), and these should be explored; but this should not deter focused efforts toward access for groups with long histories of exclusion and racialized oppression in this country. The impact of federal, state, or local legislation and funding may modify an individual institution's approach to addressing disparities in access to medical education.

## Abbreviations Used

CBSA: core-based statistical area
URiM: underrepresented in medicine
